# Upfront brain radiotherapy improves intracranial progression-free survival but not overall survival in lung adenocarcinoma patients with brain metastases: a retrospective, single-institutional analysis from China

**DOI:** 10.7150/jca.64335

**Published:** 2022-01-01

**Authors:** Aijun Jiang, Meng Ni, Li Li, Fen Zhao, Yuanhu Yao, Xin Ding, Qingxi Yu, Longzhen Zhang, Shuanghu Yuan

**Affiliations:** 1 Department of Radiation Oncology, Shandong Cancer Hospital, Shandong University, Jinan, 250117, Shandong, China.; 2 Department of Radiation Oncology, Qingdao University Medical College Affiliated Yantai Yuhuangding Hospital, Yantai, 264000, Shandong, China.; 3 Department of Radiation Oncology, Affiliated Hospital of Xuzhou Medical University, Xuzhou, 221002, Jiangsu, China.

**Keywords:** brain metastases, lung adenocarcinoma, epidermal growth factor receptor, tyrosine kinase inhibitor, brain radiotherapy

## Abstract

**Aims:** The optimal timing of brain radiotherapy (BRT) for lung adenocarcinoma patients with brain metastases (BM) remains controversial. In this retrospective study, we performed a retrospective review to investigate the differential benefit of upfront versus deferred BRT for lung adenocarcinoma patients with BM.

**Methods:** A total of 354 lung adenocarcinoma patients with BM treated in the Affiliated Cancer Hospital of Shandong University met the inclusion criteria for the study. Patients were divided into two groups: upfront BRT and deferred BRT. Intracranial progression-free survival (PFS) and overall survival (OS) were measured from the date of brain metastases. Subgroup analyses according to gene mutation status were also performed.

**Results:** Among the entire cohort, the median intracranial PFS with upfront BRT (16.3 months) was longer than that with deferred BRT (11.3 months, p=0.001). However, the median OS did not differ significantly between patients who received upfront BRT and deferred BRT (27.6 and 31.5 months, respectively, p=0.813). Subgroup analyses indicated that upfront BRT yielded a significantly longer intracranial PFS than deferred BRT (p=0.003) for patients without EGFR (19 or 21) mutation. In both subgroups, the median OS showed no significant difference between upfront BRT and deferred BRT.

**Conclusion:** This single-institutional retrospective study showed that in lung adenocarcinoma patients with brain metastases, upfront BRT was associated with a significantly longer intracranial PFS but not improvement in OS compared with deferred BRT. Considering the neurocognitive toxicities of BRT previously reported in the literature, deferred BRT might be considered as an acceptable therapeutic option for the treatment of patients with lung adenocarcinoma and BM.

## Introduction

Non-small cell lung cancer (NSCLC) accounts for about 87% of all lung cancer cases 1 and 20-40% of NSCLC patients will develop brain metastases (BM) 2, 3. The proportion of adenocarcinoma among NSCLC cases has continued to increase in recent years 1. In Asians, ~50% of lung adenocarcinomas harbor epidermal growth factor receptor (EGFR) mutations 4. EGFR mutation status is highly concordant between primary NSCLC and corresponding BM 5. Chemotherapy for such BM is not an effective treatment because of the existence of the blood-brain-barrier (BBB) 6. For decades whole brain radiotherapy (WBRT) has been considered the standard care for patients with BM, which is associated with an overall response rate of 60%, a 6-month disease control rate of 50% and a median overall survival (OS) of 4-6 months 7-9.

During the past decade, the advancement of EGFR tyrosine kinase inhibitors (EGFR-TKIs) has revolutionarily transformed the landscape of treatment and prognosis of advanced lung adenocarcinoma patients 10. EGFR-TKIs were demonstrated to be safe and significantly efficacious in EGFR mutated NSCLC patients with BM, leading to a median progression-free survival of 14.5 months, a median OS of 21.9 months 11 and intracranial disease response rates of 75-88% 12-14. Among patients with previously untreated advanced NSCLC with EGFR mutation, osimertinib yielded a longer OS of 38.6 months than a comparator EGFR-TKI (31.8 months) 15. EGFR TKIs are recommended as first line systemic therapy for patients with metastatic NSCLC harboring EGFR activating mutations 16, 17.

Interestingly, a recent study demonstrated that patients with EGFR-mutated NSCLC are more sensitive to RT 18. It also has been reported that combination treatment with brain radiotherapy (BRT) and EGFR-TKIs presents superior response rate and disease control rate compared with BRT or TKI alone 19, 20. However, the retrospective study by Magnuson et al 21 demonstrated inferior OS with deferral of BRT. Another study reported that upfront BRT followed by TKI therapy may be an appropriate initial management approach for EGFR-mutant NSCLC patients with BM 22.

However, in contrast to the conclusions of Magnuson et al, a retrospective analysis demonstrated that the addition of WBRT to EGFR-TKIs, compared with TKIs alone, did not improve the intracranial progression-free survival (PFS) and led to a worse OS in NSCLC with EGFR mutation and BM 23. A study in China also suggested that upfront WBRT can be safely delayed in EGFR-mutant lung cancer with BM 24. In the European Society of Medical Oncology guidelines for metastatic NSCLC, delay of upfront WBRT is considered in NSCLC patients with minimal and asymptomatic BM.

Thus, there is no consensus on the management of patients with lung adenocarcinoma and BM, and specifically, the optimal timing of BRT has yet to be determined. In the present retrospective study, we investigated the differential benefit of upfront versus deferred BRT for lung adenocarcinoma patients with BM. We also performed subgroup analyses according to gene mutation status.

## Materials and methods

### Study design and patient selection

This study was conducted in compliance with the Declaration of Helsinki and approved by the Ethics Committee of the Affiliated Cancer Hospital of Shandong University (SDTHEC: 201611004). Through a review of hospital records, we identified 776 lung adenocarcinoma patients with BM treated at the Affiliated Cancer Hospital of Shandong University between June 1, 2013 and October 31, 2016. The eligibility criteria for this study were: (1) lung adenocarcinoma with BM, and (2) treatment with linear accelerator-based BRT. The exclusion criteria were: (1) upfront use of a TKI before BM diagnosis; (2) failure to complete the BRT plan; (3) meningeal metastases; (4) surgical resection or stereotactic radiosurgery (SRS); (5) presence of other primary tumors; (6) history of BRT; and (7) < 3 months of loss to follow-up. The included patients were divided into two groups: upfront BRT and deferred BRT. Upfront BRT was defined as BRT within 2 months after diagnosis of BM. Tumor response was assessed every 2-3 months according to response evaluation criteria in solid tumors (RECIST) 1.1 25 by contrast-enhanced magnetic resonance imaging (MRI) or computed tomography (CT).

The last date of follow-up was March 31, 2018. The following variables were collected at BM diagnosis: (1) age; (2) gender; (3) smoking status; (4) Karnofsky performance status (KPS) ; (5) histology; (6) gene mutation status; (7) extracranial metastases; (8) size of largest BM; (9) number of BMs; (10) symptoms of BMs; (11) radiotherapy mode; and (12) treatments. A disease-specific Graded Prognostic Assessment (DS-GPA) was calculated. The radiotherapy modes were divided into three groups: WBRT, LBRT (local brain radiotherapy) and WBRT + LBRT. The gross tumor area radiotherapy was named as LBRT. The equivalent dose in 2 Gy/fraction (EQD2) of WBRT was calculated to be 32.5-50 Gy based on the LQ model. The accumulated LBRT dose was 50-60Gy with 2-4Gy per fraction and one fraction per day. When subgroup analyses, patients with ALK, ROS-1, or c-MET mutation were excluded. The dates of initial cancer diagnosis, BM diagnosis, BRT, systemic treatments, intracranial progression, most recent follow-up, and death were recorded. Intracranial PFS was defined as the time from BM diagnosis to intracranial progression, death, or last follow-up. OS was defined as the time from BM diagnosis to death or last follow-up.

### Statistical analysis

Statistical comparisons between two groups were performed in SPSS, version 21.0 (SPSS Inc., Chicago, IL, USA). Comparisons of clinical characteristics between the upfront and deferred BRT groups were performed using the chi-square test. Kaplan-Meier analysis was used to estimate intracranial PFS and OS and generate surviva1 curves, and log-rank testing was used to assess differences between the groups. Multivariable Cox proportional hazards analysis were performed according to backward stepwise regression. Differences were considered to be statistically significant if p<0.05.

## Results

### Patient characteristics

After application of the inclusion and exclusion criteria, a total of 354 patients were identified for survival analyses (273 who received upfront BRT and 81 who received deferred BRT). The patients' demographic and clinical characteristics are summarized in Table 1. The median follow-up for all patients was 23.0 months (range, 0.6-113.6 months). The median age at BM diagnosis for all patients was 57 years (range, 27-78 years). The median ages at BM diagnosis for the upfront BRT and deferred BRT groups were 57 and 55 years, respectively. Additionally, 188 (53%) patients were male, and 89 patients (25%) were smokers. In total, 91 patients (25.7%) displayed EGFR (19 or 21) mutation, 5 patients (1.4%) displayed ALK+ mutation, 77 patients (21.8%) displayed no EGFR (19 or 21) or ALK gene mutation, and 181 patients (51.1%) did not undergo genetic testing. Among the patients with a positive gene mutation, 92.7% patients (89/96) received targeted therapy. However, 42.0% patients (76/181) with unknown gene mutation status and 35.1% patients (27/77) without EGFR gene mutation also received targeted therapy.

Among all 354 patients, 168 patients (47.5%) received WBRT, 54 (15.3%) received LBRT, and 132 (37.3%) received both WBRT and LBRT. The median maximum diameter of the BMs was 15.5 mm. Overall, 52.5% of patients (186/354) had BM at primary diagnosis. Patients who received upfront BRT were more likely to have symptomatic BM (64.1% vs. 37%; p<0.001) and were more likely to have BMs >1 cm (72.9% vs. 44.4%; p<0.001). Patients who received upfront BRT were less likely to have extracranial metastases (56% vs. 72.8%; p=0.007) and more likely to receive LBRT and WBRT (41.8% vs. 22.2%; p=0.006). No differences were observed between the two groups with respect to age, sex, Eastern Cooperative Oncology Group (ECOG) performance status, smoking status, number of BMs, gene mutation, or DS-GPA at the time of BM diagnosis.

### Survival outcomes for the entire cohort

**Figure 1:**
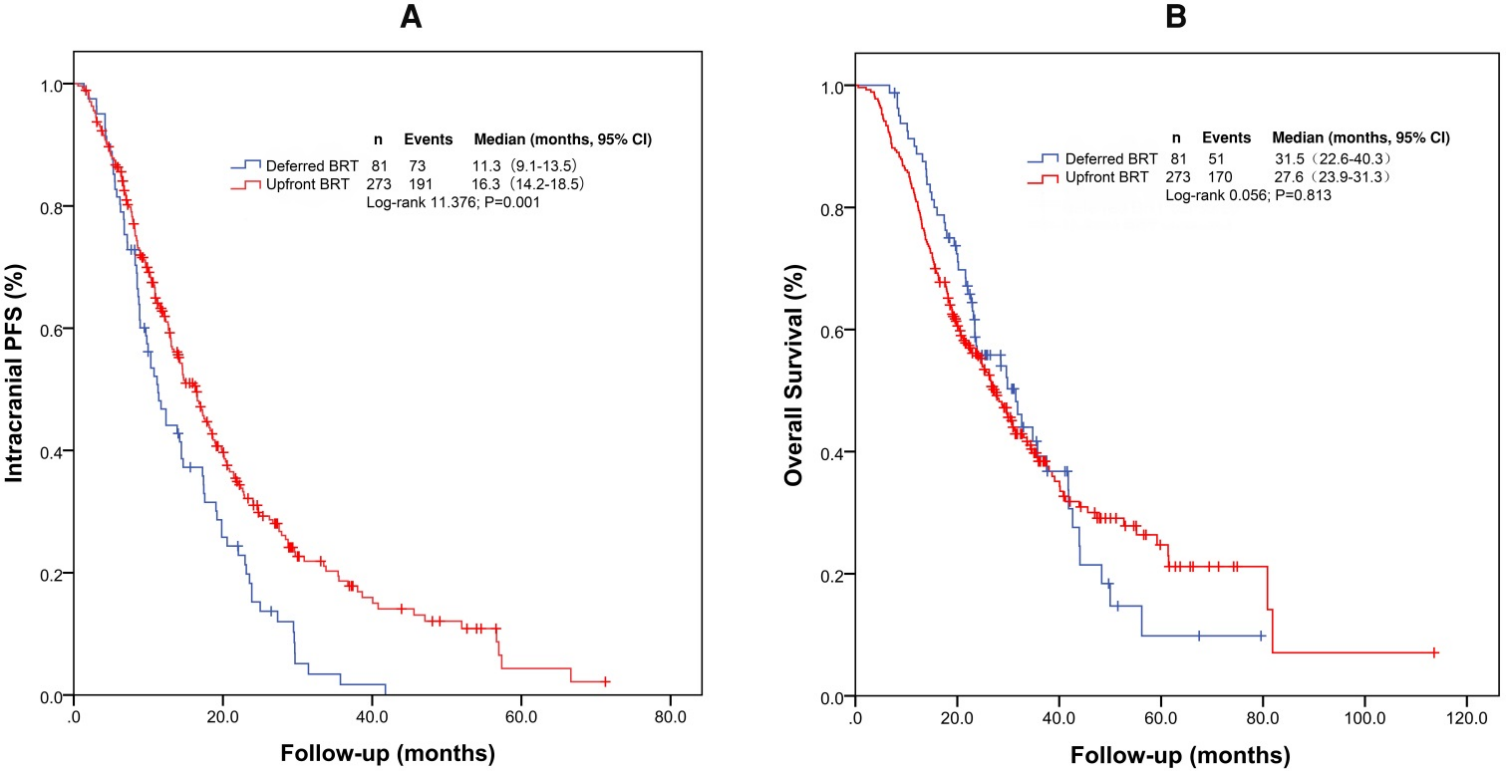
Kaplan-Meier survival curves of intracranial PFS (A) and OS (B) for the entire cohort. Abbreviations: BRT: brain radiotherapy; PFS: progression-free survival; OS: overall survival.

For the entire cohort, the median intracranial PFS and OS were 14.6 months (95% confidence interval [CI]: 12.4-16.7 months) and 28.2 months (95% CI: 24.7-31.7 months), respectively. The median intracranial PFS durations with upfront BRT and deferred BRT were 16.3 months (95% CI: 14.2-18.5 months) and 11.3 months (95% CI: 9.1-13.5 months), respectively (Figure 1A and 1B). The median intracranial PFS with upfront BRT was significantly longer than that with deferred BRT (log-rank p=0.001; Table 2). The impact of upfront BRT on intracranial PFS remained significant on multivariable analysis (p=0.003, hazard ratio [HR]=0.662[0.503-0.871]). Interestingly, the median OS with upfront BRT (27.6 months, 95% CI: 23.9-31.3 months) appeared to be shorter than that with deferred BRT (31.5 months, 95% CI: 22.6-40.3 months), but the difference was not significant (log rank p=0.813; Table 3).

### Subgroup analyses of patients with EGFR (19 or 21) mutation

According to the gene mutation status, we performed further analyses by dividing the patients into three groups: EGFR (19 or 21) mutation group (positive), no EGFR (19 or 21) mutation group (negative), and no genetic testing group (unknown). Patients with ALK (5 patients) were excluded. For patients with EGFR (19 or 21) mutation, the median intracranial PFS and OS were 17.1 months (95% CI: 13.7-20.5 months) and 34.4 months (95% CI: 28.0-40.9 months), respectively. With upfront BRT and deferred BRT, the median intracranial PFS and OS were 18.0 vs 14.2 months (χ^2^= 2.222, p=0.136) and 34.4 vs 34.8 months (χ^2^=0.006, p=0.938), respectively. Univariate analysis indicated that upfront BRT and WBRT+LBRT were associated with intracranial PFS, but the differences were not significant. Upfront BRT was not significantly associated with OS according to the univariate and multivariate analyses (Supplementary Table 4 and 5).

### Subgroup analyses for patients without EGFR (19 or 21) mutation

For patients without EGFR (19 or 21) mutation, the median intracranial PFS and OS were 10.8 (95% CI: 9.3-12.4) and 23.4 months (95% CI: 19.0-27.9), respectively. Upfront BRT in these patients provided a longer median intracranial PFS than deferred BRT (12.3 vs 8.5 months, χ^2^=9.682, p=0.002). However, no difference in the median OS (23.4 vs. 23.4 months, χ^2^=0.137, p=0.711) was observed between the patients who received upfront BRT and deferred BRT. On multivariate analysis, only upfront BRT yielded a longer intracranial PFS than deferred BRT (p=0.003, HR=0.412[0.231-0.733]). However, no correlation between upfront BRT and OS was found on univariate and multivariate analyses. Multivariate analysis identified only the number of BMs as significantly associated with OS (p=0.051, HR=0.570[0.324-1.002]) (Supplementary Table 6 and 7).

## Discussion

Among NSCLC subtypes, adenocarcinoma is more prone to BM, and the prognosis and treatment strategy for lung adenocarcinoma patients are different from those of patients with other lung cancer types, especially for patients with EGFR gene mutations. The upfront BRT strategy has advantages and disadvantages. Although previous studies indicated that upfront BRT may be associated with better local control and longer OS, use of TKIs alone initially avoids the side effects of BRT. Two small randomized studies reported that the timing of WBRT did not influence OS in unselected NSCLC patients with BM receiving chemotherapy 26, 27. Upfront SRS did not improve OS in oligo-brain metastases NSCLC patients compared with upfront chemotherapy 28. The QUARTZ study 29 indicated that WBRT provided little additional clinically significant benefit for BM patients with a KPS of <70. Use of an EGFR-TKI alone for BM in EGFR-mutant lung cancer patients showed outcomes comparable to those who received upfront RT followed by EGFR-TKI therapy 30. The BRAIN study also suggested that upfront BRT can be safely delayed in EGFR-mutant lung cancer patients with BM 24. Thus, to date, the value and timing of BRT for patients with BM from lung adenocarcinoma have remained controversial, especially for EGFR-mutant patients.

To the best of our knowledge, this study was the first retrospective analysis from China to compare survival outcomes with upfront and deferred BRT in patients with BM from lung adenocarcinoma. The targeted population in this study was lung adenocarcinoma patients with BM regardless of number of BMs, which was different from the population of the BRAIN study 24. Among the entire cohort, the median intracranial PFS was longer in patients who received upfront BRT than in those who received deferred BRT. However, in our subgroup analysis according to gene mutation status, intracranial PFS no longer significantly differed with upfront versus deferred BRT for patients with EGFR (19 or 21) mutation. Additionally, no significant differences in OS were observed between upfront and deferred BRT in both subgroups. Therefore, upfront BRT may offer an intracranial PFS benefit in patients with BM from lung adenocarcinoma, especially in patients without EGFR (19 or 21) mutation, but cannot provide an additional OS benefit.

Several qualitative reviews have suggested that TKI therapy may be used first before BRT in EGFR+ NSCLC patients with asymptomatic BM 31-34. However, Magnuson et al 21 reported that the use of upfront EGFR-TKI and deferral of RT was associated with inferior OS in patients with EGFR-mutant NSCLC who developed BM. For EGFR-mutant BM patients, our subgroup results showed that upfront BRT may provide a longer intracranial PFS compared to deferred BRT, but the difference was not significant. On the other hand, upfront BRT was not significantly associated with OS according to the univariate and multivariate analysis.

In the present study, upfront BRT was defined as BRT within 2 months after diagnosis of BM, which was not consistent with the definition used in several previous studies 21, 23, 30. Although SRS is recommended by guidelines as a standard radiotherapy method for patients with 1-3 BMs, it is not available for most RT units in China. Therefore, patients treated with SRS were excluded from the present study. Due to the lack of randomization, the presence of bias was inevitable. In upfront BRT group, patients were more likely to have symptomatic BM, BMs >1 cm and no extracranial metastases. Patients who received deferred BRT were less likely to receive WBRT + LBRT. All of these may affect the intracranial PFS and OS of the patients. Furthermore, the data from the subgroup analysis comparing the two treatment groups were insufficient due to the small number of patients treated with deferred BRT. The quality of evidence supporting these findings is low. In addition, the adverse events, quality of life and cognitive function could not be assessed due to the lack of clinical data. Moreover, the patients involved in the present study were all Chinese; hence, further investigations in patients of other races are required.

## Conclusions

In summary, this single-institutional, retrospective study showed that in patients with lung adenocarcinoma and BM, upfront BRT was associated with a significantly longer intracranial PFS, especially in patients without EGFR (19 or 21) mutation. No difference in OS was observed between patients treated with upfront BRT versus deferred BRT. Considering the neurocognitive toxicities previously reported with upfront BRT, deferred BRT might be considered as an acceptable therapeutic option for the treatment of patients with lung adenocarcinoma and BMs.

## Supplementary Material

Supplementary tables 4-13 (numbering continued from tables within the paper).

## Figures and Tables

**Figure 2:**
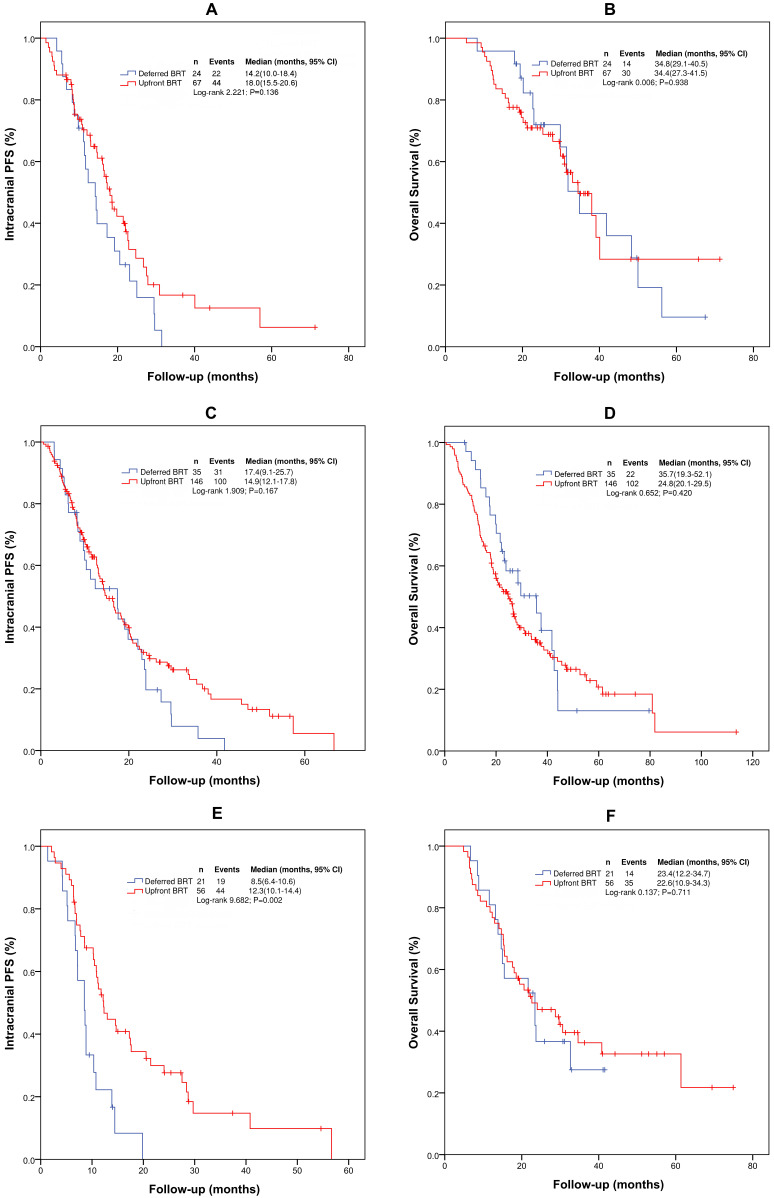
Kaplan-Meier survival curves for intracranial PFS and OS in patient subgroups. (A) Intracranial PFS for patients with EGFR (19 or 21) mutation; (B) OS for patients with EGFR (19 or 21) mutation; (C) Intracranial PFS for patients without genetic testing results; (D) OS for patients without genetic testing results; (E) Intracranial PFS for patients without EGFR (19 or 21) mutation; (F) OS for patients without EGFR (19 or 21) mutation. Abbreviations: BRT: brain radiotherapy; PFS: progression-free survival; OS: overall survival.

**Table 1:** Characteristics of 354 lung adenocarcinoma patients with BM.

	Upfront BRT(n=273)		Deferred BRT(n=81)			
Characteristic	n	%	n	%	χ^2^	p
Age					0.133	0.716
≤60 years	176	64.5	54	66.7		
>60 years	97	35.5	27	33.3		
Gender					2.327	0.127
Male	151	55.3	37	45.7		
Female	122	44.7	44	54.3		
Smoking status					0.295	0.587
Current/former	90	33.0	24	29.6		
Never	183	67.0	57	70.4		
Symptomatic BM					18.773	0.000
Yes	175	64.1	30	37.0		
No	98	35.9	51	63.0		
No. of BM					0.756	0.385
1-3	164	60.1	53	65.4		
>3	109	39.9	28	34.6		
Size of largest BM					22.656	0.000
≤1cm	74	27.1	45	55.6		
>1cm	199	72.9	36	44.4		
Gene mutation					2.673	0.263
Positive	71	26.0	25	30.9		
Negative	56	20.5	21	25.9		
Unknown	146	53.5	35	43.2		
Extracranial metastases						
Yes	153	56.0	59	72.8	7.335	0.007
No	120	44.0	22	27.2		
DS-GPA					0.123	0.726
0-2	166	60.8	51	63.0		
2.5-4	107	39.2	30	37.0		
BRT mode					10.210	0.006
WBRT	120	44.0	48	59.3		
LBRT	39	14.2	15	18.5		
WBRT + LBRT	114	41.8	18	22.2		

Abbreviations: BM: brain metastases; DS-GPA: Diagnosis-Specific Graded Prognostic Assessment; BRT: brain radiotherapy; WBRT: whole brain radiotherapy; LBRT: local brain radiotherapy.

**Table 2:** Univariable and multivariable analyses of covariables associated with intracranial PFS in lung adenocarcinoma patients with BM.

	Univariable		Mutivariable		
**Variable**	**χ^2^**	**p**	**HR**	**95% CI**	**p**
Upfront BRT vs deferred BRT	11.376	0.001	0.662	0.503-0.871	0.003
Age (≤60 vs >60 years)	0.865	0.352			
Gender (male vs female)	2.244	0.134			
Smoking status (never vs current/former)	1.313	0.252			
Symptom (yes vs no)	1.475	0.225			
No. of BM (1-3 vs >3)	1.435	0.231			
Size of largest BM (≤1 vs >1cm)	0.229	0.632			
Gene mutation					
Positive vs negative	4.051	0.044			
Positive vs unknown	0.003	0.958			
Negative vs unknown	4.018	0.045			
Extracranial metastases (yes vs no)	12.466	0.000			
DS-GPA (2.5-4 vs 0-2)	13.380	0.000	0.659	0.510-0.851	0.001
BRT mode					
LBRT vs WBRT	0.224	0.636			
LBRT + WBRT vs WBRT	3.885	0.049			
LBRT + WBRT vs LBRT	1.070	0.301			

Abbreviations: BM: brain metastases; DS-GPA: Diagnosis-Specific Graded Prognostic Assessment; BRT: brain radiotherapy; WBRT: whole brain radiotherapy; LBRT: local brain radiotherapy.

**Table 3:** Univariable and multivariable analyses of covariables associated with OS in lung adenocarcinoma patients with BM.

	Univariable		Mutivariable		
**Variable**	**χ^2^**	**p**	**HR**	**95% CI**	**p**
upfront BRT vs deferred BRT	0.056	0.813			
Age (≤60 vs >60 years)	0.027	0.869			
Gender (male vs female)	4.259	0.039			
Smoking status (never vs current/former)	4.517	0.034	0.651	0.489-0.865	0.003
Symptom (yes vs no)	0.532	0.466			
No. of BM (1-3 vs >3)	7.141	0.008	0.678	0.515-0.892	0.006
Size of largest BM (≤1 vs >1cm)	3.318	0.069	0.730	0.546-0.976	0.034
Gene mutation					
Positive vs negative	3.869	0.049	0.660	0.442-0.985	0.042
Positive vs unknown	4.300	0.038	0.637	0.455-0.893	0.009
Negative vs unknown	0.012	0.914			
Extracranial metastases (yes vs no)	14.194	0.000	1.749	1.314-2.330	0.000
DS-GPA (2.5-4 vs 0-2)	9.740	0.002			
BRT mode					
LBRT vs WBRT	0.679	0.410			
LBRT + WBRT vs WBRT	3.198	0.074			
LBRT + WBRT vs LBRT	0.185	0.667			

Abbreviations: BM: brain metastases; DS-GPA: Diagnosis-Specific Graded Prognostic Assessment; BRT: brain radiotherapy; WBRT: whole brain radiotherapy; LBRT: local brain radiotherapy.
