# Supplementary material for: Upfront brain radiotherapy improves intracranial progression-free survival but not overall survival in lung adenocarcinoma patients with brain metastases: a retrospective, single-institutional analysis from China
Source: J Cancer. 2022 Jan 1;13(2):602–9. doi: 10.7150/jca.64335 (PMC8771513; doi:10.7150/jca.64335)
Supplement: Supplementary file 1 — Supplementary tables 4-13 (numbering continued from tables within the paper). [file jcav13p0602s1.pdf]

Table 4. Univariable and multivariable analyses of covariables associated with intracranial PFS in EGFR (19 or 21) mutation-positive lung adenocarcinoma patients with BM

	**Univariable**		**Mutivariable**		
**Variable**	**χ^2**	**p**	**HR**	**95% CI**	**p**
upfront BRT vs deferred BRT	2.221	0.136			
Age（≤60 vs ＞60 years）	0.777	0.378			
Gender (male vs female)	0.004	0.950			
Smoking status (never vs current/former)	0.157	0.692			
Symptom (yes vs no)	0.412	0.521			
No. of BM (1–3 vs ＞3)	0.153	0.695			
Size of largest BM (≤1 vs ＞1cm)	0.352	0.553			
Extracranial metastases (yes vs no)	0.036	0.850			
DS-GPA (2.5–4 vs 0–2)	0.003	0.955			
BRT mode					
LBRT vs WBRT	0.195	0.659			
LBRT + WBRT vs WBRT	1.794	0.180			
LBRT + WBRT vs LBRT	1.842	0.175			

Abbreviations: BM, brain metastases; DS-GPA, Diagnosis-Specific Graded Prognostic Assessment; BRT, brain radiotherapy; WBRT, whole brain radiotherapy; LBRT, local brain radiotherapy.

Table 5. Univariable and multivariable analyses of covariables associated with OS in EGFR (19 or 21) mutation-positive lung adenocarcinoma patients with BM

	**Univariable**		**Mutivariable**		
**Variable**	χ^2	**p**	**HR**	**95% CI**	**p**
upfront BRT vs deferred BRT	0.006	0.938			
Age（≤60 vs ＞60 years）	0.707	0.401			
Gender (male vs female)	1.467	0.226			
Smoking status (never vs current/former)	0.662	0.416			
Symptom (yes vs no)	2.018	0.155			
No. of BM (1–3 vs ＞3)	1.923	0.166			
Size of largest BM (≤1 vs ＞1cm)	2.112	0.146			
Extracranial metastases (yes vs no)	5.950	0.015			
DS-GPA (2.5–4 vs 0–2)	1.368	0.242			
BRT mode					
LBRT vs WBRT	0.003	0.954			
LBRT + WBRT vs WBRT	1.915	0.166			
LBRT + WBRT vs LBRT	1.643	0.200			

Abbreviations: BM, brain metastases; DS-GPA, Diagnosis-Specific Graded Prognostic Assessment; BRT, brain radiotherapy; WBRT, whole brain radiotherapy; LBRT, local brain radiotherapy.

Table 6. Univariable and multivariable analyses of covariables associated with intracranial PFS in EGFR (19 or 21) mutation-negative lung adenocarcinoma patients with BM

	Univariable		Mutivariable		
**Variable**	χ^2	p	HR	95% CI	p
upfront BRT vs deferred BRT	9.682	0.002	0.412	0.231–0.733	0.003
Age（≤60 vs ＞60 years）	1.149	0.284			
Gender (male vs female)	0.137	0.711			
Smoking status (never vs current/former)	0.580	0.446			
Symptom (yes vs no)	3.580	0.058			
No. of BM (1–3 vs ＞3)	1.142	0.285			
Size of largest BM (≤1 vs ＞1cm)	0.046	0.830			
Extracranial metastases (yes vs no)	0.528	0.467			
DS–GPA (2.5–4 vs 0–2)	0.010	0.919			
BRT mode					
LBRT vs WBRT	0.006	0.939			
LBRT + WBRT vs WBRT	0.041	0.839			
LBRT + WBRT vs LBRT	0.052	0.820			

Abbreviations: BM, brain metastases; DS-GPA, Diagnosis-Specific Graded Prognostic Assessment; BRT, brain radiotherapy; WBRT, whole brain radiotherapy; LBRT, local brain radiotherapy.

Table 7. Univariable and multivariable analyses of covariables associated with OS in EGFR (19 or 21) mutation-negative lung adenocarcinoma patients with BM

	Univariable		Mutivariable		
**Variable**	χ^2	**p**	**HR**	**95% CI**	**p**
upfront BRT vs deferred BRT	0.137	0.711			
Age（≤60 vs ＞60 years）	0.085	0.770			
Gender (male vs female)	0.886	0.347			
Smoking status (never vs current/former)	1.296	0.255			
Symptom (yes vs no)	0.242	0.623			
No. of BM (1–3 vs ＞3)	3.917	0.048	0.570	0.324–1.002	0.051
Size of largest BM (≤1 vs ＞1cm)	0.001	0.971			
Extracranial metastases (yes vs no)	0.358	0.550			
DS-GPA (2.5–4 vs 0–2)	0.313	0.252			
BRT mode					
LBRT vs WBRT	0.000	0.993			
LBRT + WBRT vs WBRT	0.487	0.485			
LBRT + WBRT vs LBRT	0.658	0.417			

Abbreviations: BM, brain metastases; DS-GPA, Diagnosis-Specific Graded Prognostic Assessment; BRT, brain radiotherapy; WBRT, whole brain radiotherapy; LBRT, local brain radiotherapy.

Table 8. Median intracranial PFS in lung adenocarcinoma patients with BM

**Variable**	Median intracranial PFS (95% CI)
upfront BRT vs deferred BRT	16.3 (14.2–18.5) vs 11.3 (9.1–13.5)
Age（≤60 vs ＞60 years）	14.1 (12.7–15.4) vs 17.4 (14.0–20.9)
Gender (male vs female)	13.4 (11.2–15.7) vs 16.5（14.2–18.8)
Smoking status (never vs current/former)	16.1 (13.4–18.8) vs 14.0 (11.1–16.9)
Symptom (yes vs no)	14.6 (12.2–17.0) vs 14.4 (10.3–18.5)
No. of BM (1–3 vs ＞3)	16.1 (13.5–18.7) vs 13.4 (11.3–15.7)
Size of largest BM (≤1 vs ＞1cm)	16.8 (14.5–19.1) vs 13.4 (11.7–15.1)
Gene mutation	18.9 (14.9–22.9) vs 12.7 (11.0–14.4)
Positive vs negative	17.3 (13.8–20.7) vs 10.8 (9.3–12.4)
Positive vs unknown	17.3 (13.8–20.7) vs 16.3 (13.4–19.3)
Negative vs unknown	10.8 (9.3–12.4) vs 16.3 (13.4–19.3)
Extracranial metastases (yes vs no)	12.7 (11.0–14.4) vs 18.9 (14.9–22.9)
DS-GPA (2.5–4 vs 0–2)	12.9 (11.2–14.7) vs 18.9 (15.1–22.7)
BRT mode	
LBRT vs WBRT	11.8 (8.1–15.5) vs 14.2 (12.3–16.1)
LBRT + WBRT vs WBRT	16.6 (12.8–20.3) vs 14.2 (12.3–16.1)
LBRT + WBRT vs LBRT	16.6 (12.8–20.3) vs 11.8 (8.1–15.5)

Abbreviations: BM, brain metastases; DS-GPA, Diagnosis-Specific Graded Prognostic Assessment; BRT, brain radiotherapy; WBRT, whole brain radiotherapy; LBRT, local brain radiotherapy.

Table 9. Median OS in lung adenocarcinoma patients with BM

**Variable**	Median OS (95% CI)
upfront BRT vs deferred BRT	27.6 (23.9–31.3) vs 31.5 (22.6–40.3)
Age（≤60 vs ＞60 years）	27.1 (22.5–31.7) vs 29.7 (23.2–36.2)
Gender (male vs female)	25.6 (20.5–30.8) vs 29.9 (24.8–34.9)
Smoking status (never vs current/former)	29.9 (25.4–34.3） vs 22.7 (16.1–29.3)
Symptom (yes vs no)	28.9 (23.9–33.9) vs 24.8 (19.2–30.4)
No. of BM (1–3 vs ＞3)	31.2 (26.7–35.8) vs 22.7 (18.0–27.4)
Size of largest BM (≤1 vs ＞1cm)	31.8 (23.9–39.8) vs 26.5 (22.8–30.2)
Gene mutation	
Positive vs negative	34.4 (29.9–39.0) vs 23.4 (19.0–27.9)
Positive vs unknown	34.4 (29.9–39.0) vs 26.1 (22.1–30.2)
Negative vs unknown	23.4 (19.0–27.9) vs 26.1 (22.1–30.2)
Extracranial metastases (yes vs no)	23.4 (20.8–26.0) vs 35.7 (31.5–40.0)
DS-GPA (2.5–4 vs 0–2)	22.6 (19.4–25.9) vs 33.8 (28.5–39.1)
BRT mode	
LBRT vs WBRT	23.9 (16.9–30.8) vs 28.5 (24.1–33.0)
LBRT + WBRT vs WBRT	30.9 (25.6–36.1) vs 28.5 (24.1–33.0)
LBRT + WBRT vs LBRT	30.9 (25.6–36.1) vs 23.9 (16.9–30.8)

Abbreviations: BM, brain metastases; DS-GPA, Diagnosis-Specific Graded Prognostic Assessment; BRT, brain radiotherapy; WBRT, whole brain radiotherapy; LBRT, local brain radiotherapy.

Table 10. Median intracranial PFS in EGFR (19 or 21) mutation-positive lung adenocarcinoma patients with BM

**Variable**	Median intracranial PFS (95% CI)
upfront BRT vs deferred BRT	18.0 (15.5–20.6) vs 14.2 (10.0–18.4)
Age（≤60 vs ＞60 years）	14.7 (11.3–18.1) vs 19.2 (16.8–21.6)
Gender (male vs female)	14.6 (6.2–23.0) vs 17.3 (15.9–18.7)
Smoking status (never vs current/former)	16.3 (13.5–19.2) vs 18.6 (10.5–26.7)
Symptom (yes vs no)	16.3 (13.3–19.3) vs 18.0 (13.0–23.1)
No. of BM (1–3 vs ＞3)	17.3 (14.9–19.7) vs 14.6 (10.2–19.0)
Size of largest BM (≤1 vs ＞1cm)	16.5 (11.9–21.2) vs 17.1 (13.1–21.1)
Extracranial metastases (yes vs no)	16.5 (13.3–19.8) vs 17.1 (9.1–25.1)
DS-GPA (2.5–4 vs 0–2)	16.5 (12.7–20.4) vs 17.1 (11.3–22.9)
BRT mode	
LBRT vs WBRT	11.7 (5.9–17.5) vs 14.6 (11.7–17.5)
LBRT + WBRT vs WBRT	22.7 (17.4–27.9) vs 14.6 (11.7–17.5)
LBRT + WBRT vs LBRT	22.7 (17.4–27.9) vs 11.7 (5.9–17.5)

Abbreviations: BM, brain metastases; DS-GPA, Diagnosis-Specific Graded Prognostic Assessment; BRT, brain radiotherapy; WBRT, whole brain radiotherapy; LBRT, local brain radiotherapy.

Table 11. Median OS in EGFR (19 or 21) mutation-positive lung adenocarcinoma patients with BM

**Variable**	Median OS (95% CI)
upfront BRT vs deferred BRT	34.4 (27.3–41.5) vs 34.8 (29.1–40.5)
Age (≤60 vs > 60 years)	31.8 (27.9–35.8) vs 39.1 (33.2–45.0)
Gender (male vs female)	40.1 (23.3–56.8) vs 31.5 (25.6–37.3)
Smoking status (never vs current/former)	34.4 (27.8–41.1) vs 34.8 (19.4–50.2)
Symptom (yes vs no)	33.0 (26.6–39.6) vs 41.8 (26.6–57.1)
No. of BM (1–3 vs > 3)	40.1 (28.0–52.1) vs 33.0 (21.6–44.4)
Size of largest BM (≤1 vs > 1cm)	48.3 (26.2–70.5) vs 33.0 (28.6–37.5)
Extracranial metastases (yes vs no)	31.4 (29.3–33.4) vs 48.3 (37.8–58.9)
DS-GPA (2.5–4 vs 0–2)	34.4 (29.3–39.6) vs 40.1 (29.0–51.1)
BRT mode	
LBRT vs WBRT	11.7 (5.9–17.5) vs 14.6 (11.7–17.5)
LBRT + WBRT vs WBRT	22.7 (17.4–27.9) vs 14.6 (11.7–17.5)
LBRT + WBRT vs LBRT	22.7 (17.4–27.9) vs 11.7 (5.9–17.5)

Abbreviations: BM, brain metastases; DS-GPA, Diagnosis-Specific Graded Prognostic Assessment; BRT, brain radiotherapy; WBRT, whole brain radiotherapy; LBRT, local brain radiotherapy.

Table 12. Median intracranial PFS in EGFR (19 or 21) mutation-negative lung adenocarcinoma patients with BM

**Variable**	Median intracranial PFS (95% CI)
upfront BRT vs deferred BRT	12.3 (10.1–14.4) vs 8.5 (6.4–10.6)
Age（≤60 vs ＞60 years）	10.8 (7.0–14.5) vs 10.8 (8.9–12.8)
Gender (male vs female)	10.9 (9.2–12.6) vs 10.8 (6.7–14.8)
Smoking status (never vs current/former)	10.8 (8.5–13.2) vs 10.9 (6.9–14.9)
Symptom (yes vs no)	12.3 (9.9–1.6) vs 8.5 (5.5–11.6)
No. of BM (1–3 vs ＞3)	10.3 (7.7–12.9) vs 12.3 (7.7–16.9)
Size of largest BM (≤1 vs ＞1cm)	10.8 (5.2–16.5) vs 10.9 (9.8–12.0)
Extracranial metastases (yes vs no)	10.3 (7.8–12.9) vs 11.8 (9.4–14.1)
DS-GPA (2.5–4 vs 0–2)	10.9 (8.8–13.0) vs 10.5 (6.6–14.4)
BRT mode	
LBRT vs WBRT	10.9 (9.5–12.3) vs 8.8 (6.9–10.7)
LBRT + WBRT vs WBRT	12.2 (9.8–14.6) vs 8.8 (6.9–10.7)
LBRT + WBRT vs LBRT	12.2 (9.8–14.6) vs 10.9 (9.5–12.3)

Abbreviations: BM, brain metastases; DS-GPA, Diagnosis-Specific Graded Prognostic Assessment; BRT, brain radiotherapy; WBRT, whole brain radiotherapy; LBRT, local brain radiotherapy.

Table 13. Median OS in EGFR (19 or 21) mutation-negative lung adenocarcinoma patients with BM

**Variable**	Median OS (95% CI)
upfront BRT vs deferred BRT	22.6 (10.9–34.3) vs 23.4 (12.2–34.7)
Age（≤60 vs ＞60 years）	22.6 (17.8–27.5) vs 28.7 (10.8–46.7)
Gender (male vs female)	22.6 (17.1–28.2) vs 23.4 (11.7–35.1)
Smoking status (never vs current/former)	23.4 (9.2–37.6) vs 21.7 (14.7–28.7)
Symptom (yes vs no)	24.0 (14.0–34.1) vs 21.6 (15.7–2.7)
No. of BM (1–3 vs ＞3)	30.6 (19.5–41.7) vs 16.2 (8.9–23.5)
Size of largest BM (≤1 vs ＞1cm)	23.4 (12.5–34.4) vs 21.9 (15.0–28.8)
Extracranial metastases (yes vs no)	22.6 (16.8–28.5) vs 24.0 (9.7–38.3)
DS-GPA (2.5–4 vs 0–2)	18.6 (11.4–25.7) vs 29.7 (18.3–41.1)
BRT mode	
LBRT vs WBRT	10.9 (9.5–12.3) vs 8.8 (6.9–10.7)
LBRT + WBRT vs WBRT	12.2 (9.8–14.6) vs 8.8 (6.9–10.7)
LBRT + WBRT vs LBRT	12.2 (9.8–14.6) vs 10.9 (9.5–12.3)

Abbreviations: BM, brain metastases; DS-GPA, Diagnosis-Specific Graded Prognostic Assessment; BRT, brain radiotherapy; WBRT, whole brain radiotherapy; LBRT, local brain radiotherapy.
